# Design of electric and remote operating vehicles battery carrier by using small aluminum closed-cell foam blocks shielded by aluminum tubes

**DOI:** 10.1038/s41598-026-39720-z

**Published:** 2026-03-17

**Authors:** Mohamed H. Dadoura, Ahmed I. Farahat, Zaid Ali Al-Saady, M. R. Taha, Ramadan N. Elshaer

**Affiliations:** 1 Tabbin Institute for Metallurgical Studies, Cairo, Egypt; 2 Central Metallurgical Research and Development Institute, Cairo, Egypt; 3 Zubair Field Operating Division (ZFOD), Basra, Iraq; 4 Mechanical Department, Faculty of Engineering, Cairo University, Cairo, Egypt; 5 Tabbin Institute for Metallurgical Studies, Cairo, Egypt

**Keywords:** Energy absorption, Corrosion, Heat transfer, Design, Sandwich panel, ACCFBs, Cooling time, Engineering, Materials science

## Abstract

Aluminum closed-cell foam blocks (ACCFBs) are small blocks of foam shielded with small aluminum (Al) tubes developed to enhance the energy absorption of Al foam in limited volumes, such as one cubic inch. It is designed to overcome the problems of the high cost of production, maintenance, and heat insulation properties of foam sheets. Al blocks ideas quoted from human and animal bone parts, and it is designed to absorb energy laterally. This work presents the designs of remote operating vehicles (ROVs) and electric vehicles (EVs) battery carriers, constructed from a cube block of ACCFBs and two Al sheets. The carrier design is based on the properties of Al foam blocks. It is designed to withstand temperatures up to 120 °C. The carrier idea relies on replacing large sheets of Al foam with small, distributed blocks. It has good energy absorption, and at the same time, it helps solve the problems of harness design for crossing wires in narrow areas, such as in ROVs. The results show that the ROVs battery carrier made from the Al sandwich panel (AFS) needs 900 s to transfer 120 °C from the upper sheet to the lower sheet, while the carrier made from ACCFBs with the same volume needs 40 s only. It can also bear a load of up to 0.8 kN and has a working strength **δ**_working_: 1.093 MPa. The EVs battery carrier can bear a load of 117 kN (about 25 times the battery weight) at yield strength (0.45 MPa), and it has a **δ**_working_: 0.516 MPa. The calculated total time of cooling for the conduction and forced convection for both the ROVs and EVs carriers at a cooling temperature of 25 °C and at air velocity 1 m/s were 4:31 min and 32:25 min, and at 2 m/s were 3:15 min and 20:41 min, respectively, at a summer working temperature of 40 °C.

## Introduction

The Al foam sheets have common problems in applications that concluded in the cost of fabrication, sheet maintenance, and heat insulation properties of foam sheets. Al closed-cell foam blocks (ACCFBs) consist of small parts of foam shielded with Al tubes, small parts, and in some types. It is reinforced by small tubes too, not exceeding 30% of the block height. The idea of blocks was quoted from the skeleton bones of humans and animals. ACCFB patterns are a group of blocks arranged to achieve the required properties with low volume or area compared to the Al foam to avoid the problems of big Al foam parts. They consist of similar blocks or different types of blocks according to achieving compound properties like energy absorption with good heat transfer. Three properties of Al/Al foam blocks (ACCFBs) should be considered during the pattern design: energy absorption of the used block versus the Al foam block, corrosion, and heat transfer of blocks. The main aim of the design is to simplify the fabrications and calculations of pattern energy absorption, where amateurs, technicians, and engineers will be able to make patterns from traditional market components for both foam and shielding tubes. This study simplifies the calculations of energy absorption of the small blocks at lateral impacts. This theory is quoted from the natural movement of some bones in the skeleton, like the lower jaw and hand fingers. Hence, the hand-in punch position presents a pattern designed to receive impacts. Also, hands, when leaning on a wall or table, present a flat pattern affected by quasi-static stress^1–3^.

The idea of foam blocks is the enhancement of the energy absorption of Al Foam with small parts by shielding it with Al tubes or simply improving the ability of energy absorption for small blocks with limited volume. This idea aims to solve the problems faced by big sheets used to absorb energy, which work as heat insulators and have high costs in fabrication and maintenance for non-uniform shapes. So, by reducing volume, small blocks will be easy to replace defective blocks as expendable material, then reduce the cost of production and maintenance, and allow heat transfer to occur for the part of its shielding as a connector between the heated surface connected to the object and the lower surface facing the ambient. ACCFBs in commonly designed to use traditional materials that exist in the market. It consists of tube shapes, like circular or square shapes^4,5^, and Al foam small blocks. Material selection is important for design properties, so to select the foam, the base material, relative density, pore size, pore structure type (closed, open, or mixed), and densification strength should be identified^6,7^. Also, to select shieling and inner tubes, material, shape, dimensions, and yield strength should be recognized.

**Fig. 1:**
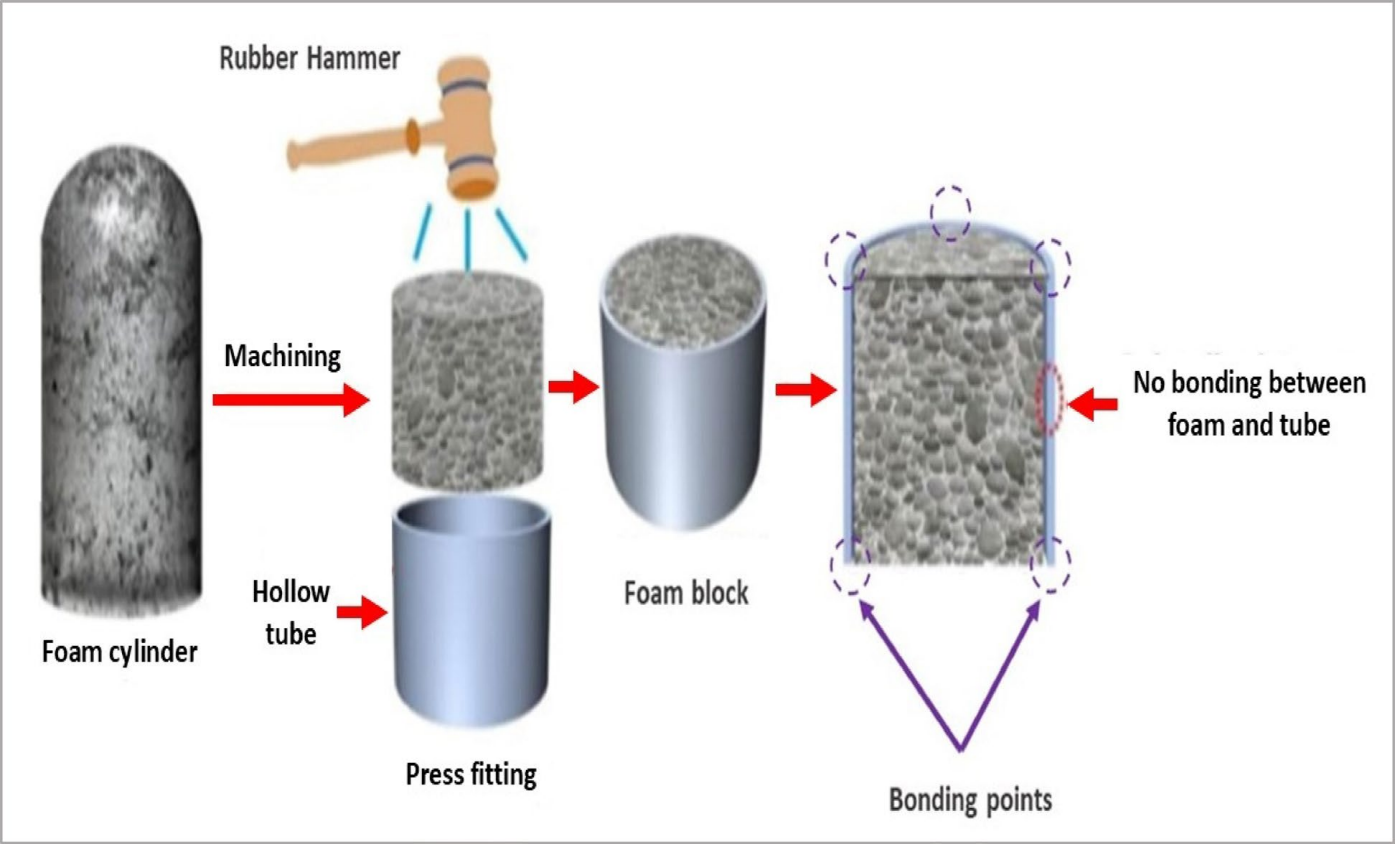
Schematic illustration of the fabrication for Al foam blocks by machining method^8^.

For applying the fabrication processes, the Al foam small blocks should be cut from plates have the same properties, and it’s preferable to follow the same manufacturer. The simple method of producing foam blocks is the machining method which depends on cutting and forming foam parts by using suitable tools (e.g. wire cut or diamond saw for cutting foam, saw for tubes, files, drill, and rubber hammer) and after preparing foam samples and tube parts with the required dimensions assembling it by soft hammering as illustrated in Fig. 1. After that bonded it together by adhesive metallic resin from both ends of the block by four points apart 90° angle for circular shapes. And four points at the midpoint of the segments of rectangular and square shapes. Two points are sufficient for adhesive inner tubes on both sides of ACCFBs^8,9^.

**Fig. 2:**
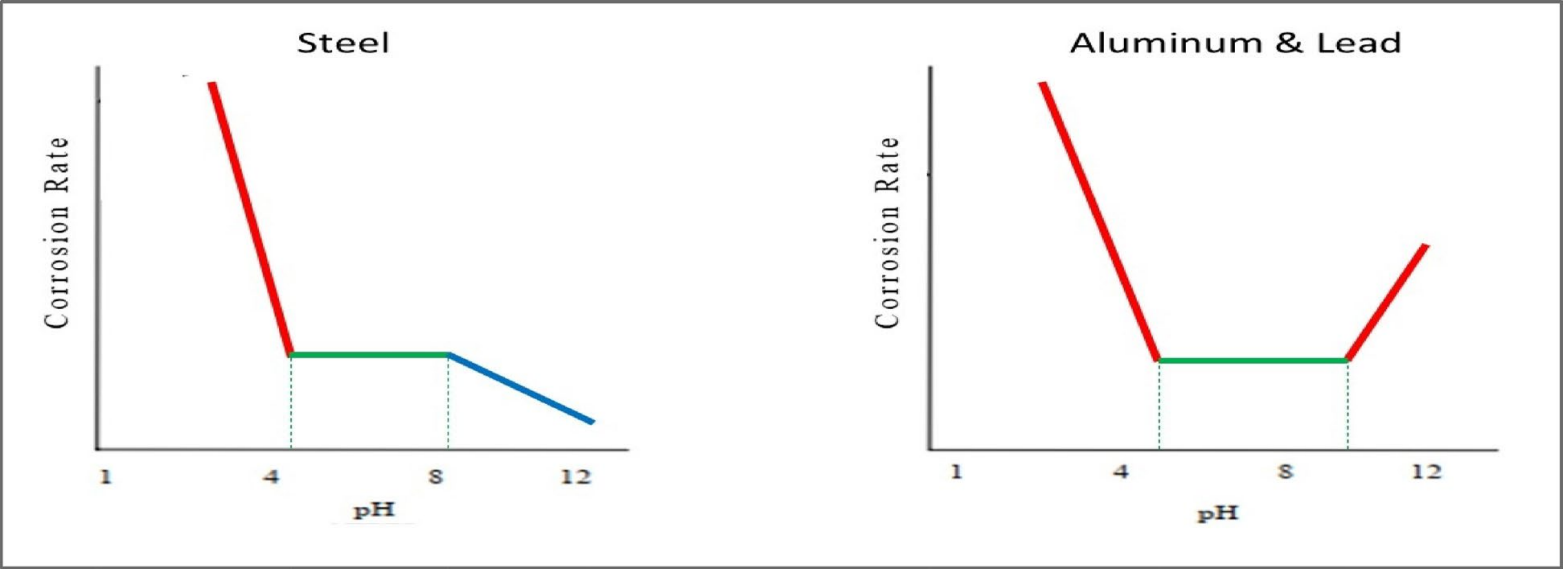
Effect of pH scale on the corrosion rate of steel and aluminium, and lead^9^.

Al classified as an amphoteric material, can react with strong acidic and alkalis. It has stability between 4 and 8.5 pH, where the corrosion rate is fairly independent of pH, and the metal is covered with an Al_2_O_3_ protective oxide film according to its nature. This is because the protective oxide film on the surface of these metals is dissolved in the strongest acids and alkalis, and the metals corrode^10,11^. Figure 2 exposes the curves for the stability of Al versus pH by comparing them to steel. Corrosion potential of Al-1.0 V^8^ and Al foam-1.16 V^12^ in seawater with salinity 3.5% NaCl vs Ref Ag/AgCl. Hence, Al passivates at chloride concentrations less than 1446 ppm, which equals 0.26 wt% NaCl or 2600 ppm^13^. Galvanic corrosion occurs when the two metals in contact have a potential difference of at least 100 mV^14^. So, corrosion is suspected to occur for ACCFBs where the difference in corrosion potential between Al and Al foam is about -160 mV.

Al has been used for cooling. It has good thermal properties and high thermal conductivity, while Al Foam is used as a heatproof (Insulator) material^15^. Al is used in most applications, like the automotive and communication industries, while closed-cell Al foam works as a heat insulator and energy absorber against impact for the same applications. Table 1 exposes the thermal properties of cast Al and Al foam^16–19^.

**Table 1:** Basic thermal properties of cast and foamed Al^16–19^.

Property	Max. operating temperature	Melting point	Specific heat	Thermal conductivity	Thermal expansion
	(°C)	(°C)	(J/kg K)	(W/m K)	(1/°C)
Cast Al	165	570	980	160	22.9 × 10^–6^
Al foam	170	560	850	12	23 × 10^–6^

## Experimental work

**Fig. 3:**
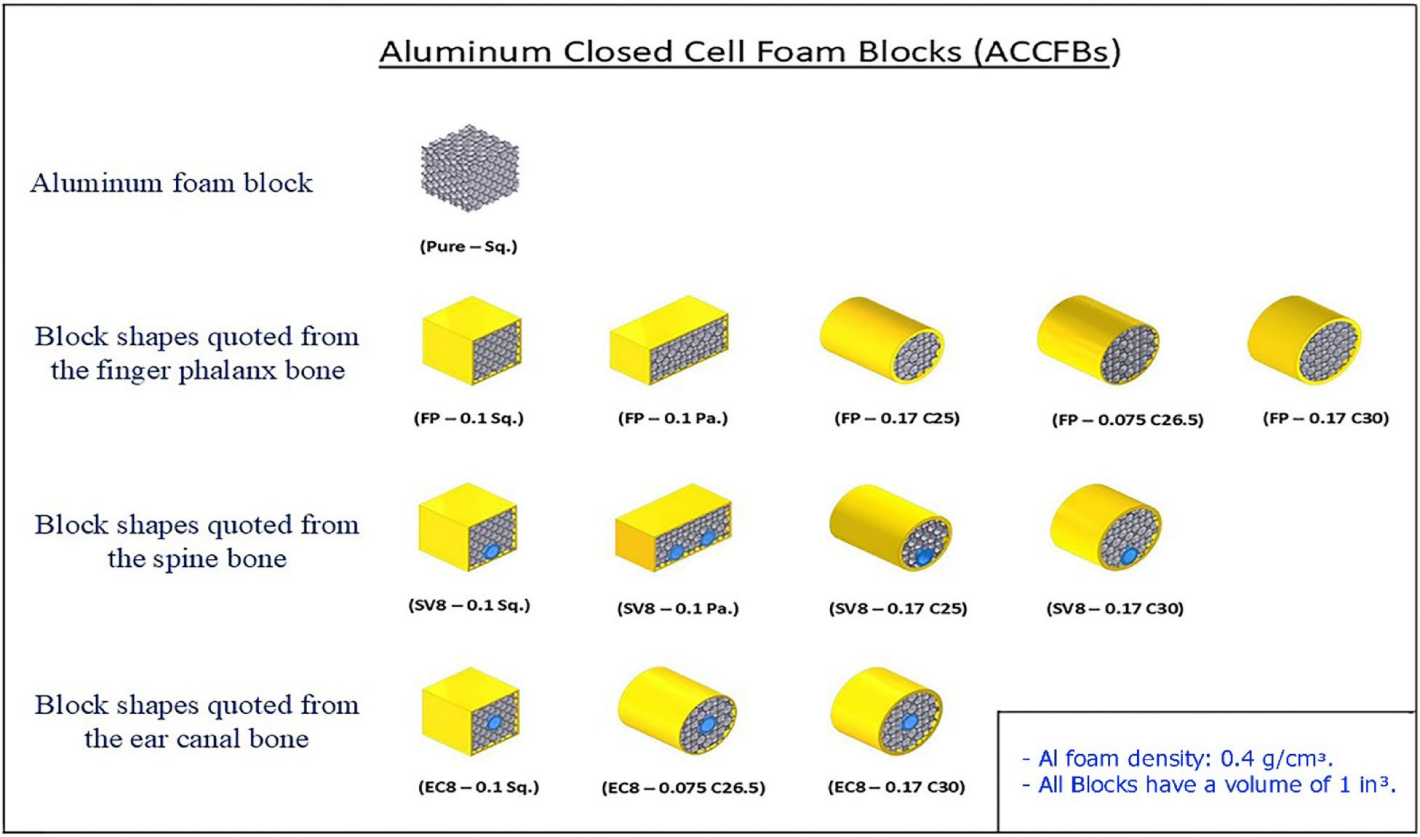
All shapes of Al closed-cell foam blocks (ACCFBs)^1^.

The tube material was chosen to be Al, with rectangular, square, and circular shapes. The material of Al tubes used in shielding foam blocks is Al 6060, as per EN 755-2 standard specifications^4^. The foam material was chosen to be closed-cell Al foam from type ALPORAS, with cell size 4 mm, relative density 14%, density 400 kg/m^3^, wall thickness 0.22 mm on average, and compressive strength 8.4 MPa at 70% strain. The volumes were selected to be one in^3^ for all blocks to compare the enhancement percentage of energy dissipation density (E_dd_) by Al foam with the same size^1^. The small volume gives blocks flexibility to shield nonuniform and complex shapes. Besides this, it is expendable, making it easy to maintain at low cost by replacing only the defective blocks in the pattern after the shielding part is exposed to impacts. Twelve blocks were designed to simulate the shapes of finger phalanx, spine, and ear canal bones. Figure 3 displays the photos of the ACCFBs.

**Fig. 4:**
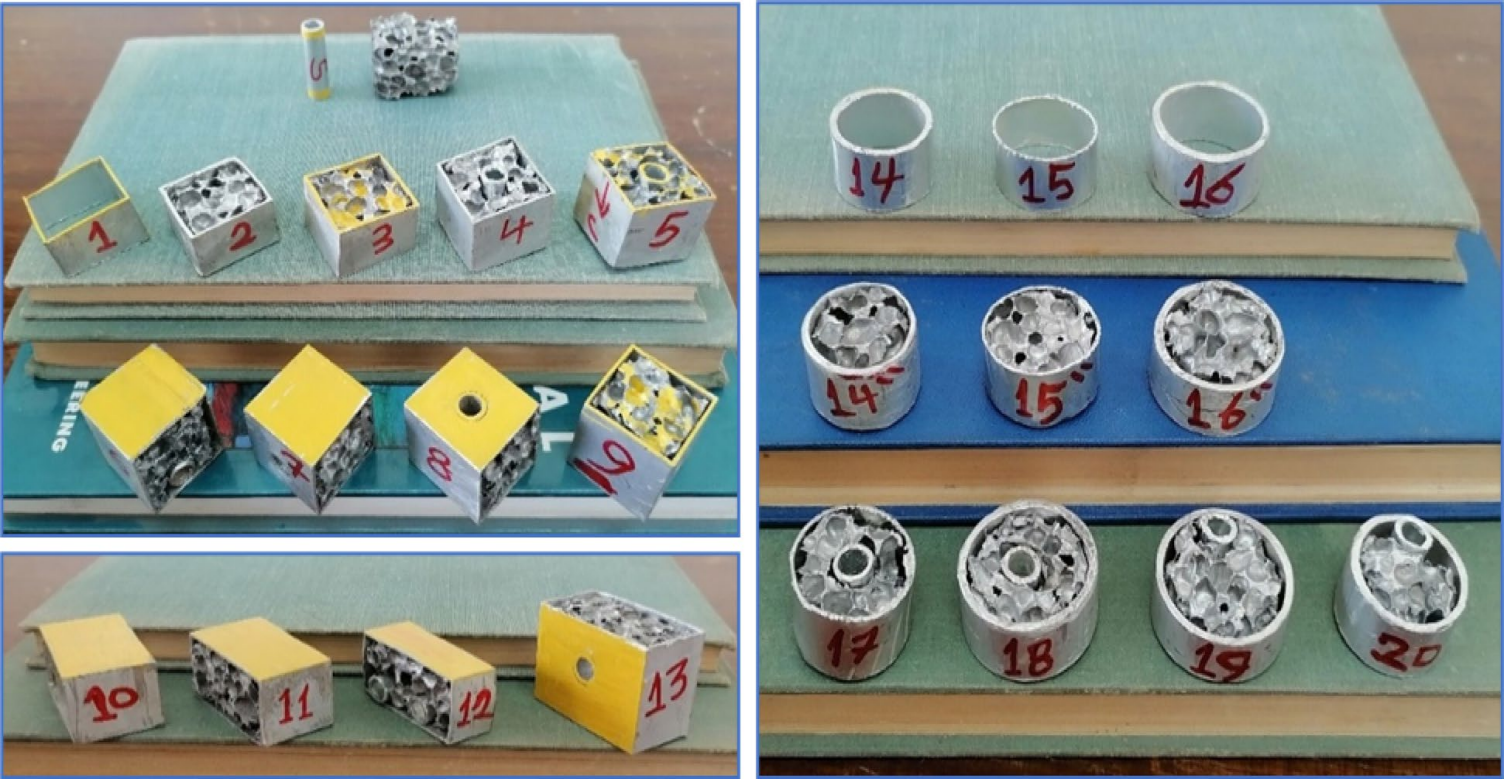
Samples of Al foam blocks with volume one in^3^.

All blocks are designed to have a volume of one in^3^ concerning their shape. There are two constraints in the selection of dimensions: the 1st is avoiding the heat isolation of foam, so dimensions must be limited to be small, and the 2nd is the ratio of block size to cell size, where the values of mechanical properties are reduced, especially strength. So, the foam block was chosen to be bigger by about 7 times bigger than the cell size to avoid boundary effects due to foam cutting and formation^5^. The actual photos of Al foam block categories quoted from Finger phalanx, Spine, and Ear canal bones are displayed in Fig. 4. All ACCFBs energy absorption results are compared with pure foam blocks to assist in the enhancement values.

Al foam’s small parts’ energy absorption is enhanced when it is shielded by Al tubes, where small parts of foam will be weak, but the hollow tubes provide the foam block with relative resistance to deformation under impacts. All blocks were exposed to lateral compression, and the improvement percentage was compared with the Al foam block. All block energy dissipation densities (E_dd_) were tested until 70% strain. The compression tests were applied where energy absorption equals the area under the stress–strain curve. E_dd_ equals the absorption energy per block volume (i.e., J/in^3^). The test conditions were compression under a universal testing machine with constant force at a 1 mm/min velocity, according to “DIN 50134:2008-10 standard^20^. Yield strength (**δ**_y_), crushing strength (**δ**_c_), and densification strength (**δ**_d_) have been calculated from the engineering stress/strain curve^1^. The selected block for ROVs and EVs battery carriers design was from type (SV8-0.1Sq), which simulates the Spine bones idea.

## Results and discussions

### Quasi-static compression test

The criteria of block selection depend on mechanical properties and energy absorption enhancement. After that, study heat transfer and cooling rate. The work environment and harness design (wire paths) should be considered during design, especially in narrow places affected by heat or intersections. The block type (SV8-0.1Sq) chosen for the application has both medium mechanical properties and energy absorption enhancement compared with other blocks quoted from the spine bone shapes. Table 2 displays the properties of the chosen block (SV8-0.1Sq) compared to the Al foam block under the quasi-static compression test.

**Table 2:** Results of mechanical properties of the SV8-0.1Sq block and the E_dd_ enhancement (ENH) percentage^1^.

Block type	Block shape	Dimensions (mm)	Mass (gm)	Quasi-static test results				
				δ_y_ (MPa)	δ_c_ (MPa)	δ_d_ (MPa)	E_dd_ (J/in^3^)	ENH (E_dd_)`
Pure foam		25 × 25 × 25	5.91	0.71	4.53	8.4	21	Base
SV8-0.1 Sq		Shell: 25 × 25 × 25 Tube: Φ = 8, t = 1.2	13.41	0.45	6.02	10	27	+ 28.5%

Material type, shape, area, and thickness are the main factors of heat transfer. So, the selection of block type should consider the main effective method for transferring heat (i.e., Conduction, Convection, Radiation). This depends on the distribution of blocks between Al panels and the block shape. Also, the position of the panel in the device and the area are exposed to fluid flow, whether it is covered or not^21^. One of the main aims of shielding a small block of Al foam with Al tubes is to enhance its ability to transfer heat, where foam is an insulating material. So, the choice of block shape has physical and mechanical constraints that should be considered. Physically, the heat transfer type in most common in ROV and EV battery carriers will be conduction at a stagnant condition (steady state) and forced convection (transient) at motion. Radiation will be negligible in calculations due to its very low amount, cause of the distances between blocks, where the distribution should consider the change in dimensions after impacts, where the block shield will expand by about 1.5 times its length. Mechanically, the dimensions and shape of the shield tube are the main factors for choosing a block, side by side with the foam shape and its reaction against lateral compression. So, there must be a balance between the thermal distribution and the mechanical properties of the block according to the nature of the movement and design of the device^22^. The block (SV8-0.1Sq) has been chosen for the design of carriers according to its good mechanical properties, besides its ability to transfer heat in a short time. Also, it’s designed to allow wires to pass from an offset small tube to avoid the intersection of cable paths in narrow places, like in ROV, and to cool it during motion by forced convection in EV Cars.

### Remotely operated vehicles (ROV) and electrical vehicles (EV) battery carriers design

The divide big shapes into small pieces gives variety and flexibility in use; there is availability to use auxiliary materials (e. g. Silicon Rubber or polyurethane memory foam -PU- with small sizes to provide some additional properties to the pattern for enhancing damping and energy absorption). Blocks and patterns can be resized or made from different materials and thicknesses, but the determination of properties should be specified by experimental work. Before designing patterns, some issues should be considered, like the working medium (Environment), thermal stresses, the velocity of an object, the value of expected impact energy, and safety factor values to suit the requirements of applications^17,22^. Patterns could be designed from similar or dissimilar blocks according to the working conditions. It can be painted or coated to avoid corrosion. It takes many forms, like sandwich panels, filled tubes, flexible tubes, and two-direction patterns in one plan and two plans^1^. Hence, two designs were presented for ROV and EV battery carriers.

#### Design of the battery carrier of remotely operated vehicles (ROVs)

**Fig. 5:**
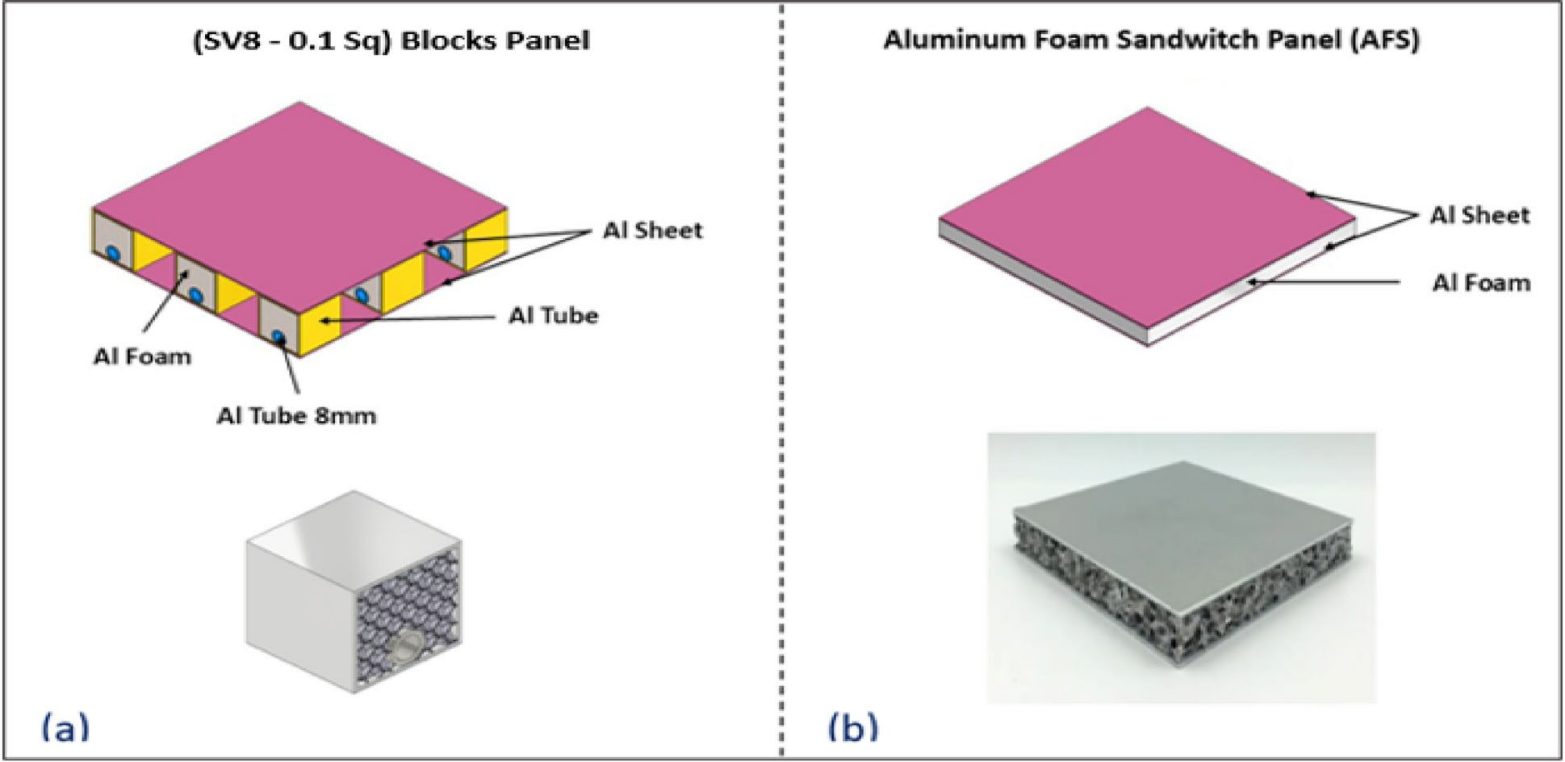
(**a**) ACCFBs pattern components, (**b**) The Al foam sandwich (AFS).

The aim of using this technique in sandwich panel design is to enhance the cooling of the sandwich panels as a carrier for batteries of electric automobiles and remotely operated vehicles (ROVs). Where Al closed-cell foam in a normal sandwich panel works as a thermal insulator. So, the work idea depends on replacing the volume of foam by suite ACCFBs with the same volume depending on the energy dissipation density property. Blocks are distributed to open paths for cooling and, at the same time, give good conductivity for heat to enhance the cooling process and energy absorption. Figure 5 displays (AFS) components and the alternative SV8-0.1Sq Blocks pattern.

- *Function/Application* Pattern designed to work as a battery carrier for batteries of small electric ROVs against impact. This one is designed to withstand temperatures up to 120°C, providing a large safety margin against the onset of rapid oxidation and creep. This ensures a long service life, as it typically operates at only 70% of its maximum operating temperature.
- *Working medium* Normal conditions. (Temperature of 25 °C).
- *Components* Two Al plates with dimensions (X = 125 mm, Y = 125 mm, Z = 1 mm) and nine square spine blocks from type (SV8-0.1Sq.) arranged to have spaces between each other with 25 mm. It can be assembled by epoxy resins (metal glue) or M2.5 set screws Fig. 5a display photo for pattern. Blocks from type (SV8-0.1 Sq.) selected to be replaced instead of a sheet of Al foam have the same properties with dimensions: (X = 125 mm, Y = 125 mm, Z = 9 mm).
- *Safety factor evaluation and selection*
  - The main purpose of considering the safety factor in design is to avoid the uncertainties in material properties, manufacturing defects, overloads, sudden failures (S.F_Material_ ≥ S.F_Design_).
  - Safety factor is defined simply as the ratio of the maximum stress that the material of the part is designed to withstand and the calculated maximum stress from the expected loads. (S.F = δ_Allowable_/δ_Working_).
  - Two principles must be distinguished when designing a safety factor: the first is for the material, which belongs to the manufacturing designer. It is used in medium and heavy loads and stress. Simply, it is equal to 1.5 or 2 times the yield or ultimate stresses for safety against sudden stresses or loads during a component’s work (i.e. Automotive, Rotary machines, transformers). The second is for design, which is related to many parameters and their weights in design, such as applied stresses, geometrical shape, and dimensions. In common for low loads S.F of material covered by S.F of design. In general, most medium loads have S.F between 2 to 4 times. For heavy loads and special applications, S.F can be greater than 4 related to applied loads and sudden failure risks^23^.
- For aluminum foam, uncertainties, such as strength, may differ slightly from the estimated value for the sample under test, as the foam cells do not have a perfectly typical shape. Sometimes it contains a slight geometrical defect or an accepted distortion during casting. This led to the actual load being higher than calculated. Besides the loading effect, conditions like impact and vibration. Degradation over time due to corrosion or fatigue. So, S.F is taken by 1.5 times for a packing material and low loads, and is equal to 2.5 to 3 times for a crash structure or automotive crash box^24^.
- *Calculation*
- By redistributing the volume of the Al sandwich panel to foam blocks according to the energy dissipation density, a foam board with a 9 mm thickness will be replaced by a Pattern containing 9 blocks from type (SV8-0.1Sq.) with energy absorption: E_dd_ = 27 J/in^3^ at 70% strain (all required data in equations are from Table 2).
  The battery weight for these dimensions will be about 50 N. This is classified low load, so select S.F to be: 1.5 times.
- 1 $$\begin{aligned} & {\text{Total energy absorption of blocks}}: \\ & {\mathrm{Ea}} = \left( {{\mathrm{No}}.{\text{ of blocks}}} \right) \, \times \, ({\mathrm{E}}_{{{\mathrm{dd}}}} ) \\ & \quad = { 9 } \times { 27 }/ \, \left( {{1}.{5}} \right) \, = { 243 }/{ 1}.{5 } = {\text{ 162 J}} \\ \end{aligned}$$
- 2 $$\begin{aligned} & {\text{Total energy absorption of Al Foam sheet}}: \\ & {\mathrm{Ea}} = \left( {{\mathrm{Vol}}.{\text{ of sheet by in}}^{{3}} } \right) \, \times \, ({\mathrm{E}}_{{{\mathrm{dd}}}} ) \\ & \quad = { 9 } \times { 21 }/ \, \left( {{1}.{5}} \right) \, = { 189 }/{ 1}.{5 } = {\text{ 126 J}} \\ \end{aligned}$$
- The maximum load (F_y p_) for the pattern at yield strength. where yield strength (**δ**_y_) is 0.45 MPa from Table 2. And the block area (A_b_) So, the allowable load that the 9 blocks can bear:
  3 $${\mathrm{F}}_{{\text{y p}}} = {\text{ A}}_{{\mathrm{b}}} {\mathrm{x }}({{\boldsymbol{\updelta}}}_{{\mathrm{y}}} ) \, /{\text{ S}}.{\text{F }} = { 9 } \times { 25 } \times { 25 } \times \, 0.{45}/{1}.{5 } = { 1687}.{\text{5 N}} \approx {1}.{7 }\;{\mathrm{kN}}$$
- 4 $$\begin{gathered} {\text{Mass of Pattern }}({\mathrm{m}}_{{\mathrm{p}}} ) = {\mathrm{No}}.{\text{ of blocks }} \times {\text{ mass of block}} + {\text{mass of Al sheets}} \hfill \\ {\mathrm{m}}_{{\mathrm{p}}} = { 9 } \times { 13}.{\text{41 gm }} + { 2 } \times { 12}.{5 } \times { 12}.{5 } \times \, 0.{1 } \times { 2}.{7 } = { 2}0{5}.0{\text{65 gm }} \approx \, 0.{\text{2 kg}}. \hfill \\ \end{gathered}$$
- Determining the maximum design load during a crush (F_crush_) for a pattern through applying the Work-Energy Theorem: The energy (i.e. kinetic or potential energy) of the impacting object must be absorbed by the work done by the crushing foam. So,
- 5 $$\begin{gathered} {\mathrm{F}}_{{{\mathrm{crush}}}} \times {\mathrm{d}} = {\mathrm{m}} {\mathrm{g}} {\mathrm{h}},{\text{where d is crushing distance}} = {\text{strain }}(\varepsilon ) \times {\text{Al foam thickness }}\left( {\mathrm{L}} \right) \hfill \\ {\mathrm{F}}_{{{\mathrm{crush}}}} \left( {\mathrm{N}} \right) \, \times \, 0.{7 } \times { 25 }\left( {{\mathrm{mm}}} \right){/1}000 \, = { 162 }\left( {\mathrm{J}} \right) \hfill \\ {\mathrm{F}}_{{{\mathrm{crush}}}} = { 162 /}0.0{175 } = {\text{ 9257 N}} \approx {9}.{\text{26 kN}} \hfill \\ \end{gathered}$$
- The allowable stress (**δ**_allowable_) is determined by dividing the impact force (F_crush_) by the total Al foam blocks areas (A_b_), to get the actual effective stress^24^.
  6 $${{\boldsymbol{\updelta}}}_{{{\mathrm{allowable}}}} = {\text{ F}}_{{{\mathrm{crush}}}} /{\mathrm{A}}_{{\mathrm{b}}} = {9257 }\left( {\mathrm{N}} \right) \, /{ 9 } \times { 25 }\left( {{\mathrm{mm}}} \right) \, \times { 25 }\left( {{\mathrm{mm}}} \right) \, = { 1}.{64}\;{\mathrm{MPa}}$$
- 7 $${\text{The working stress (}}{\mathbf{\delta }}_{{{\mathrm{working}}}} {\text{) equals the result of dividing (}}{\mathbf{\delta }}_{{{\mathrm{allowable}}}} {\text{) by S}}{\mathrm{.F}}$$
  $${{\boldsymbol{\updelta}}}_{{{\mathrm{working}}}} = { 1}.{64}/{1}.{5} = {1}.0{9}\;{\mathrm{MPa}}$$
- Note that the calculation of falling (impact) velocity and time of impact for ROV in water must consider buoyant force and drag force due to the resistance of water.

Heat transfer measurement was applied at a steady-state temperature of 120 °C for both the Al foam sandwich (AFS) with 9 mm thickness and the spine block pattern on the upper surface at ambient temperature 25 °C and stagnant Air. The pattern needed 40 s to transfer most heat by conduction between the upper and lower panels’ surfaces, while AFS needed 900 s (15 min).

**Fig. 6:**
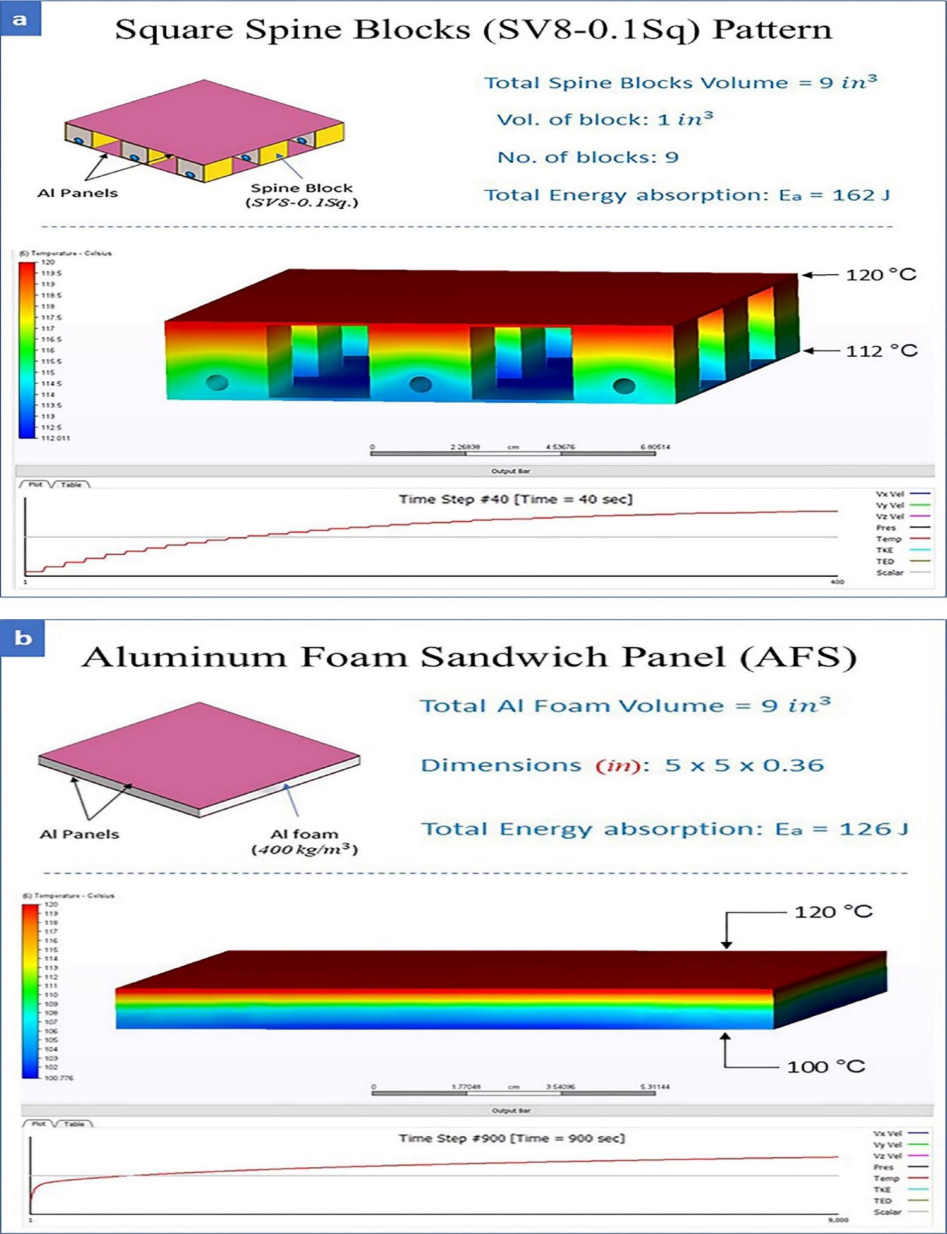
Effect of steady state heat by 120 °C on AFS and Pattern with time transient mode: (**a**) Square Spine block pattern, (**b**) AFS panel.

Hence, the energy absorption of the spine blocks (SV8-0.1Sq) pattern is better than AFS by about 29% at 70% strain. The heat transfer of the pattern is better than AFS, and its ability to cool is higher than AFS. Block panels have open paths for air flow, leading to rapid cooling. It is a good solution for passing wires or cables between tubes. It is helpful for harness design in modern remote vehicles, especially if the space between blocks is lined with insulators, such as a thin Rockwell fiber blanket. It will provide high cooling for cables and, at the same time, shield them against impacts. Remember, cables and lining shouldn’t exceed 30% of the block height to avoid damaging it after impacts. So, it will be useful for modern electric vehicles and ROVs. Most foam is designed in automotive crash boxes to absorb impacts between velocities of 4.1–8.3 m/s^25,26^. Figure 6 displays the conduction heat transfer of AFS and square spine blocks panel related to time (Calculations applied on Autodesk CFD software 2023).

#### Design of battery carrier of electric vehicles (EVs)

**Fig. 7:**
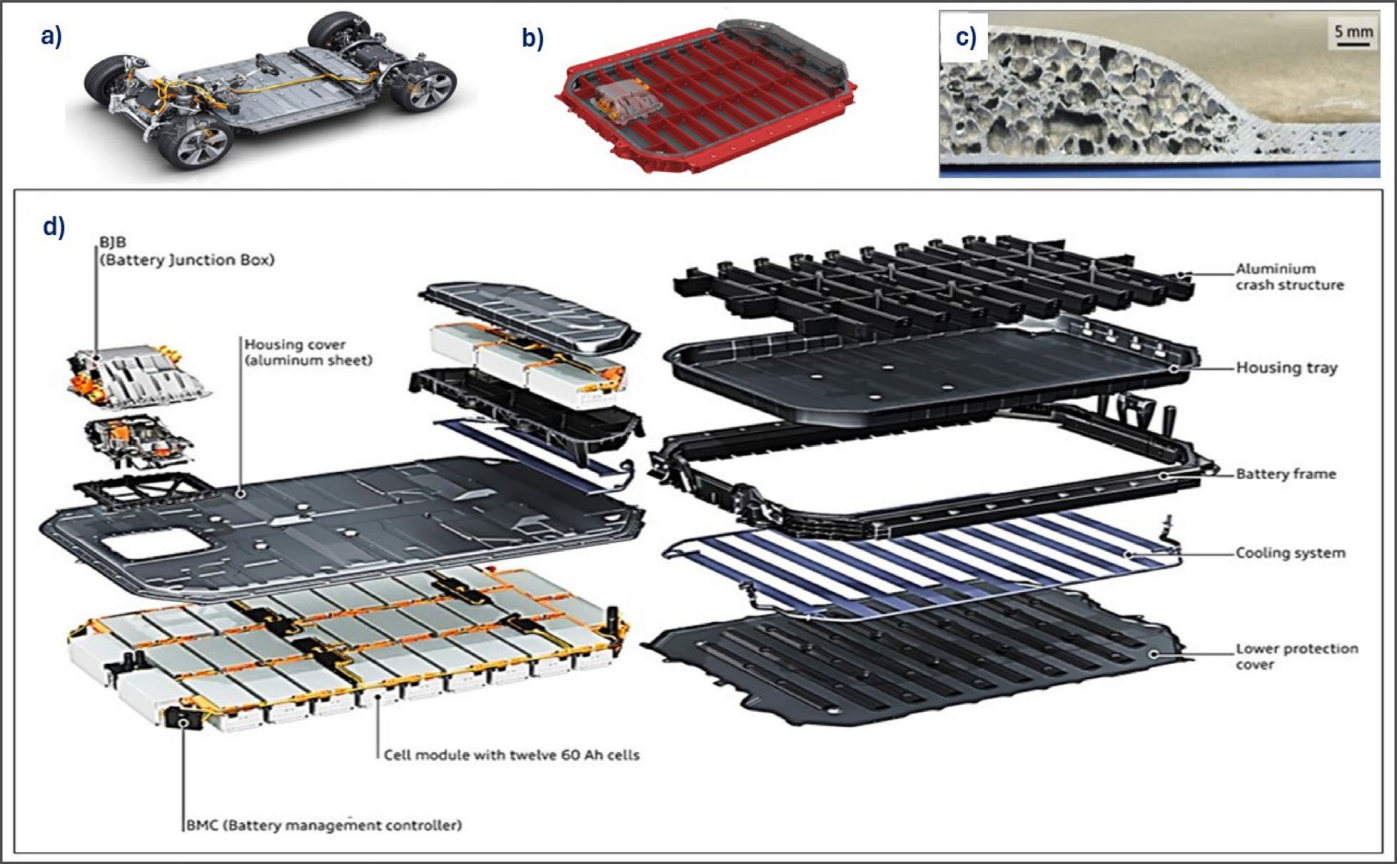
(**a**) The full electric drivetrain design for the location of the battery case and, (**b**) high voltage (HV) battery housing with crash protection structure ^29^, (**c**) AFS of under body shield with compressed transition area side to seal the panel with pack components ^27^, (**d**) Constitutive elements of the EV battery pack prototype ^28^.

Recent years have seen the development of numerous EV battery carrier models designed to improve cooling performance, in addition to their primary function of impact absorption, and solutions presented for enhanced cooling of the underbody shield by using a corrugated sheet for increasing surface area at the same time absorbing impacts under the battery cells ^27^. Although Audi is developing an extruded Al crush structure with good distribution for loads resisting longitudinal and transverse impacts ^28^. Figure 7 shows a schematic of the battery pack elements. Some metal foam companies developed a modern crash structure made of AFS supported with slots to enhance cooling.

**Fig. 8:**
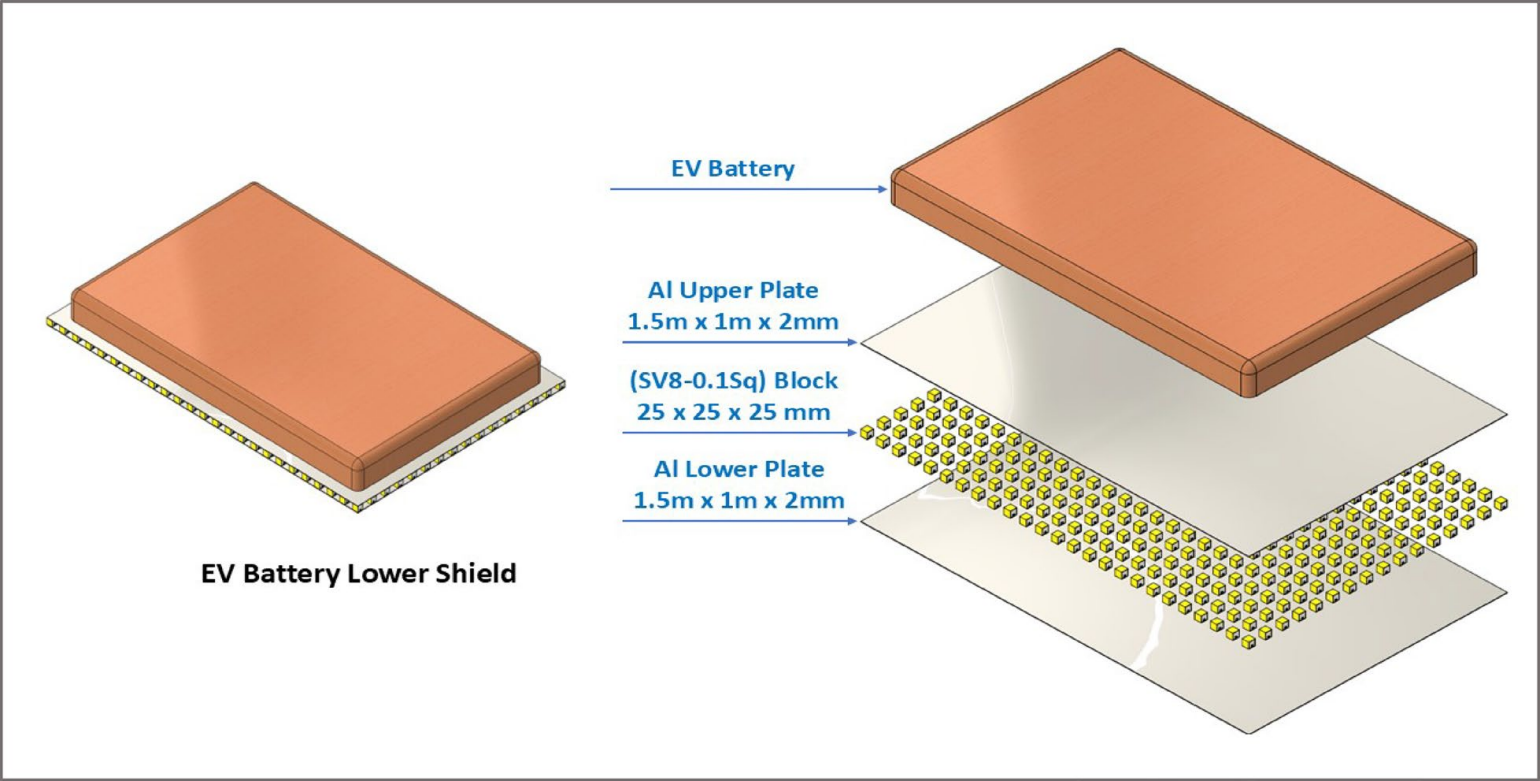
Schematic for EVs battery underbody shield consists of 2 Al sheets (1.5 m × 1 m × 2 mm) and 400 blocks from type SV8-0.1Sq. of ACCFBs.

Our research team has developed a new design for an EV battery underbody shield consisting of 400 ACCFBs (type SV8-0.1Sq.) and two Al sheets. The design can be likened to an ROV structure, but on a significantly larger scale, with a block count increased by approximately 44 times and an area increased by about 96 times. Figure 8 displays a schematic of the EV underbody shield.

- **Givens**
  EV Battery: Type: lithium-ion, Nominal Capacity 68 kWh, Usable Capacity 64.8 kWh, Dimensions (cm): 150 × 100 × 15, Mass: 450 kg. The gross weight of the automotive is 2000 kg and the ground clearance is 20 cm.
  Summer working temperature: 40 °C, Deseret working temperature: 55 °C, Ambient temperature 20 °C^30^. The maximum designed operating temperature for the carrier is 120 °C (70% of Al foam max. operating temperature) for long-term functional stability against oxidation and creep.

- **Requirement**
  Designing an EV battery carrier using ACCFBs could protect the battery against impacts up to 0.5 m in height. The working temperatures are 40, 55, and 120 °C. Also, calculate the cooling time required to cool the system at laminar flow.

- **Design and calculations of the pattern made from the square spine block**
  The carrier consists of two Al sheets with dimensions: 1.5 m *×* 1 m *×* 2 mm. The selected block type is square Spine (SV8-0.1 Sq.) and 400 distributed on a pattern with an array of 16 × 25 blocks with a space of 40 mm in the width direction and a space of 36.45 *mm* in the length direction.
- Safety Factor for automotive in the range of 2.5–3 times and for the crush box from 4–6 according to applied stresses ^24^. The design impact height of the carrier is equal to 2.25 times the ground clearance of 4 × 4 and SUV cars.
- 8 $${\text{For the battery carrier}}:{\text{ The total impact energy at }}0.{\mathrm{5height}}:{\text{ E}}_{{{\mathrm{P}},{\mathrm{bat}}}} = {\text{m g h}}$$
  where E_P,bat_ is the designed potential energy (J), m is the EV battery mass (kg), g is the acceleration of gravity (m/s^2^), and h is the height m.
  $${\mathrm{E}}_{{{\mathrm{P}},{\mathrm{bat}}}} = {45}0 \times {1}0 \times 0.{5} = {2}.{\text{25 kJ}}$$
- 9 $${\text{Total energy absorption of EV battery carrier blocks}}:{\text{ E}}_{{{\mathrm{a}},{\mathrm{blocks}}}} = \left( {{\mathrm{No}}.{\text{ of blocks}}} \right) \times \left( {{\mathrm{E}}_{{{\mathrm{dd}}}} } \right)$$
  $${\mathrm{E}}_{{{\mathrm{a}},{\mathrm{blocks}}}} = { 4}00 \times {27 } = { 1}0.{\text{8 kJ}}$$
- The impact safety factor for battery carrier could be determined as a result of dividing total absorbed energy by blocks on the designed potential energy, So:
  10 $${\mathrm{S}}.{\text{F }} = {\text{ E}}_{{{\mathrm{a}},{\mathrm{blocks}}}} /{\mathrm{E}}_{{{\mathrm{P}},{\mathrm{bat}}}} = { 4}.{8}$$
- The impact safety factor of vehicle gross weight could be determined as a result of dividing the total absorbed energy by the blocks on the designed potential energy of gross weight. So by using Eq. (8), the E_P,Gross_ = 2000 × 10 × 0.2 = 4000 J = 4 kJ
- Safety factor of vehicle gross weight. So, S.F = E_a,blocks_ /E_P,Gross_ = 10.8/4 = 2.7
- The maximum load for the carrier (F_y,blocks_) at yield strength. where yield strength (**δ**_y_) is 0.45 MPa from Table 2. So, max load carrier can bear:
  11 $${\mathrm{F}}_{{{\mathrm{y}},{\mathrm{blocks}}}} \, = \,{\mathrm{A}}_{{\mathrm{b}}} \times ({{\boldsymbol{\updelta}}}_{{\mathrm{y}}} ) \hfill \\ {\mathrm{F}}_{{{\mathrm{y,blocks}}}} {\text{ = 400 }}$$
- For determining the yield load S.F: (F_y,blocks_ /F_battery_) = (112.5 / (450 × g)) = 25
- 12 $${\text{The Mass of the carrier }}({\mathrm{m}}_{{{\mathrm{Carrier}}}} )\, = \,{\mathrm{No}}.{\text{ of blocks}} \times {\text{mass of blocks}}\, + \,{\text{mass of Al sheets}}$$
  $${\mathrm{m}}_{{{\mathrm{Carrier}}}} = \left( {{4}00 \times {13}.{\text{41 gm}}} \right) + \left( {{2} \times {15}0 \times {1}00 \times 0.{2} \times {2}.{7}} \right) = {21},{564}\;{\mathrm{gm}} \approx {21}.{5}0\;{\mathrm{kg}}$$
- For determining the maximum design load during a crush (F_crush_) for a carrier by using Eq. (5): ${\mathrm{F}}_{{{\mathrm{crush}}}} \left( {\mathrm{N}} \right) \times 0.{7} \times {25 }\left( {{\mathrm{mm}}} \right)/{1}000 = {1}0,{8}00\left( {\mathrm{J}} \right)$
  $${\mathrm{F}}_{{{\mathrm{crush}}}} = { 1}0{8}00/0.0{175 } = {\text{ 617143 N}} \approx {617}\;{\mathrm{kN}}$$
- The allowable stress (**δ**_allowable_) is determined per Eq. (6):
  $${{\boldsymbol{\updelta}}}_{{{\mathrm{allowable}}}} = {\text{ F}}_{{{\mathrm{crush}}}} /{\mathrm{A}}_{{\mathrm{b}}} = {617143 }\left( {\mathrm{N}} \right) \, /{ 4}00 \times {25 }\left( {{\mathrm{mm}}} \right) \times {25 }\left( {{\mathrm{mm}}} \right) \, = { 2}.{47}\;{\mathrm{MPa}}$$
- The working stress (**δ**_working_) equals the result of dividing (**δ**_allowable_) by S.F
  $${{\boldsymbol{\updelta}}}_{{{\mathrm{working}}}} = { 2}.{47 }/{4}.{8} = 0.{516}\;{\mathrm{MPa}}$$

#### Calculations of cooling time for the battery carriers of ROV and EV

- Assumptions ^21,31–33^:
  (1) The cooling fluid is air, (2) Spatially isothermal plate, (3) Negligible Friction, (4) The flow is laminar, (5) The convection type is forced, (6) The working temperatures are 40 °C, 55 °C, and the maximum is 120 °C,(7) Radiation is Negligible for the ROV carrier due to its small surface area, and for EV battery carrier due to the large surface. So, it will be calculated.
- Air Fluid properties are:

**Table:** 

Air density (ρ): 1.2 kg/m^3^	Specific heat capacity (C_p_): 1007 J/kg ∙ K
Dynamic viscosity (µ): 0.0000185 kg/m. s	Kinematic viscosity (ν = µ / ρ): 1.54 × 10^–5^ m^2^/s
Thermal conductivity (k): 0.026 W/m ∙ K	Thermal diffusivity (α = k/(ρ ∙ C_p_): 18.46 × 10^–6^ m^2^/s
- The formula for calculating convection heat transfer
  13 $${\mathrm{Q}}_{{{\mathrm{Conv}}}}^{ \cdot } = {\mathrm{h}} {\mathrm{A}} \Delta {\mathrm{T}}$$
  where Q˙_Conv_ is the rate of heat dissipated from the plate (Watt), h is the convective heat transfer coefficient (W/m^2^ °C), A is the surface area for heat transfer (m^2^), and ΔT is the temperature difference (°C).
- Prandtl number (Pr): is the measure of the relative thickness of the velocity and thermal boundary layer.
  14 $${\text{Pr }} = {\text{ Kinematic viscosity}}/{\text{Thermal diffusivity }} = \, \nu \, / \, \alpha$$
- Reynolds number (Re): ratio of inertia forces to viscous forces in the fluid. Re number should be less than 3.5 × 10^5^ to avoid turbulent flow.
  15 $${\mathrm{Re}} = \rho {\mathrm{U}}_{{\mathrm{m}}} {\text{L }}/ \, \mu$$
  where U_m_: is the fluid velocity, and L: is the length of the plate.
- Nusselt number (Nu): represents heat transfer enhancement through a fluid due to convection relative to conduction across the same fluid layer. It should be ≥ 0.6 for laminar flow
  16 $${\mathrm{Nu}} = 0.{66} \times {\mathrm{Re}}^{{0.{5}}} \times {\mathrm{Pr}}^{{0.{33}}}$$
- Actually, flow is not always completely laminar in many situations. illustrates the well-known example of a fluid flowing over a plate with a uniform velocity flow. A boundary layer develops close to the plate; initially, this layer is fully laminar. As the flow travels further across the plate, the boundary layer begins to flow more chaotically (i.e., a transition region) until it becomes fully turbulent ^34,35^, illustrated in Fig. 9. When calculating the Nusselt numbers, consider whether the heat is transferred from the fluid to the plate or vice versa. where the temperature is a factor considered, dependence is usually based directly on the ratio of the temperatures between fluid and wall, since for gases the temperature does not strongly influence the Prandtl number as in liquids. Hence, the critical Reynolds number Recrit, from which a transition from laminar to turbulent flow is to be expected, occurred at *Re*_*cri*_ = 10^5^, and for flow with the laminar-turbulent transition, with Reynolds numbers between 10 and 10^7^, the *average Nusselt number* is calculated for gases with the following conditions:
  17 $$\begin{gathered} {\text{Reynolds number }}\left( {{\mathrm{Re}}} \right):{ 1}0 \, < {\text{ Re }} < { 1}0^{{7}} {\text{and Prandtl number }}\left( {{\mathrm{Pr}}} \right): \, 0.{6 } < {\text{ Pr }} < { 2}000{\text{ so}}, \hfill \\ {\mathrm{Nu}}^{ * } {\text{ = Nu}} \cdot \left( {{\mathrm{T}}_{{\mathrm{f}}} /{\text{ T}}_{{\mathrm{w}}} } \right)^{{0.{12}}} \hfill \\ \end{gathered}$$

**Fig. 9:**
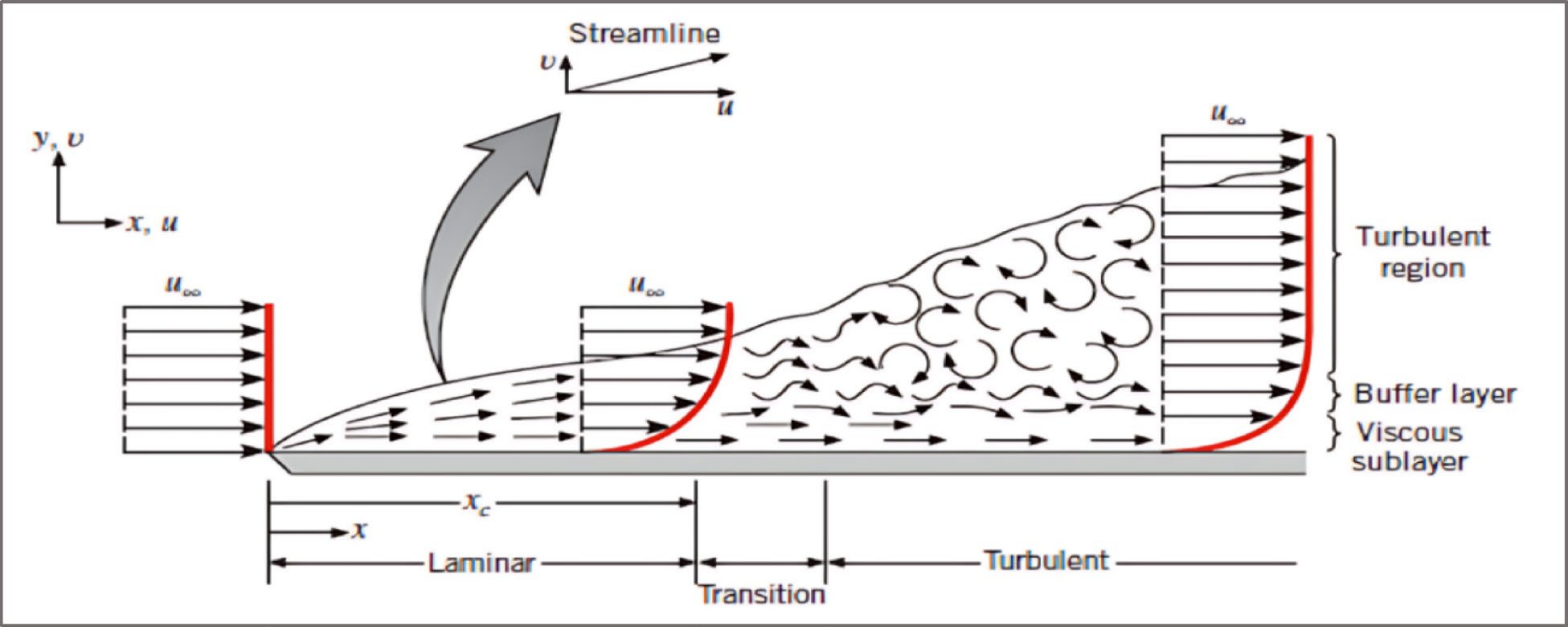
Classification of fluid flow over a flat plate ^32^.
- Heat transfer coefficient (h): defined as the reciprocal of thermal insulation. It is a quantitative characteristic of convective heat transfer between a fluid (air medium) and the surface (flat plate) flowed over by the fluid.
  18 $${\text{h }} = {\text{ Nu}}*.\left( {{\text{k }}/{\text{ L}}} \right)$$
- Fluid film temperature (T_film_): is the average temperature between the plate and ambient temperatures.
  19 $${\mathrm{T}}_{{{\mathrm{film}}}} = \, \left( {{\mathrm{T}}_{{\mathrm{p}}} + {\text{ T}}_{{\mathrm{a}}} } \right)/{2}$$
- The formula for calculating Radiation heat transfer
  20 $$\dot{Q}_{rad} = \varepsilon \cdot \sigma \cdot A_{s} (T_{s}^{4} - T_{S}^{4} )$$
  where Q˙_rad_ is the rate of heat transfer by radiation (Watt), ϵ: Emissivity of the surface (0.05 for Al), σ: Stefan-Boltzmann Constant (5.67 × ${10}^{-8}$ W/m^2^·K^4^), A_s_: is the plate surface area, ${T}_{s}:$ is Surface Temperature (K), ${T}_{sur}$: is the surrounding temperature (K) for radiation.
- *Time of cooling (t*) could be calculated from the result of the lumped capacitance equation.
  21 $${\text{Thetotal heat transferred}}:{\text{ Q}}_{{\mathrm{T}}}^{ \cdot } = {\mathrm{Q}}_{{{\mathrm{Conv}}}}^{ \cdot } + {\mathrm{Q}}_{{{\mathrm{rad}}}}^{ \cdot }$$
  For the energy balance, the rate at which the internal energy of a hot object decreases must equal the rate at which heat is lost to its surroundings by convection & radiation. Hence,
  22 $${\mathrm{Q}}_{{\mathrm{T}}}^{ \cdot } = - {\mathrm{m}}_{{\mathrm{s}}} {\mathrm{C}}_{{{\text{p }}({\mathrm{s}})}} \left( {\frac{{{\mathrm{dT}}}}{{{\mathrm{dt}}}}} \right)$$
  By substitution in equations (13, 20 and 21) find that:
  $$- msCp_{(s)} (dT/dt) = hAs(T - T_{\infty } ) + \varepsilon \sigma As(T_{s}^{4} - T_{Sur}^{4} )$$
  By rearranging this equation to estimate the required time for a temperature change *dT*:
  23 $$dt = - \left( {\left( {\frac{{m_{s} Cp_{(s)} }}{{A_{s} }}} \right)} \right) \cdot \frac{dT}{{h(T - T\infty ) + \varepsilon \sigma (T_{s}^{4} - T_{Sur}^{4} )}}$$
  So, the Integral Form for calculating the total cooling time to cool from an initial temperature to a final temperature; both sides of the equation will be integrated:
  24 $$t_{c} = - \left( {\frac{{{\uprho }_{s} {\mathrm{V}}_{s} Cp_{\left( s \right)} }}{{A_{s} }}} \right)\mathop \smallint \limits_{{T_{i} }}^{{T_{f} }} \frac{dT}{{h { }\left( {{\text{T }} - {\mathrm{T}}\infty } \right){ } + \varepsilon \sigma { }\left( {T_{s}^{4} { } - { }T_{Sur}^{4} } \right)}}$$
  where ***t***_***c***_ is the Cooling time (sec), *m*_s_ is the plate mass, ρ_s_ is the plate material density, V_s_ is the volume of the plate (m^3^), C_p (s)_ is the plate material specific heat capacity (J/kg ∙ K), *h* is the convective heat transfer coefficient (W / m^2^. K), A_s_ is the surface area for plate (m^2^), *T*_*i*_ is the plate temperature (ºC), T_∞_ is the ambient temperature, 20°C and, *T*_*f*_ is the required final cooling temperature, 25 ºC.
- The total time for cooling:
  25 $${\mathrm{t}}_{{\mathrm{T}}} = {\text{ t}}_{{{\text{conduction }} + }} {\mathrm{t}}_{{{\text{convection }} + }} {\mathrm{t}}_{{{\mathrm{radiation}}}} = {\text{ t}}_{{{\text{conduction }} + }} {\mathrm{t}}_{{\mathrm{c}}}$$

Where the conduction is a linear equation, the relationship between time and temperature difference is approximately linear due to the assumption of the stability of the heat transfer rate over time. Hence, the time is proportional to temperature differences. And cooling time till 25 °C (T_*f*_) and initial temperatures (T_*i*_) 55 °C and 40 °C could be calculated as follows for ROV battery carrier on the air:

- Initial temperature 120° to 25° : ΔT = 95°, time = 40 s.
- Initial temperature 55° to 25° : ΔT = 30°, time = (30 / 95) × 40 = 12.63 ≈ 13 s.
- Initial temperature 40° to 25° : ΔT = 15°, time = (15 / 95) × 40 = 6.32 ≈ 6 s.

For ROV battery carrier on the water:

The heat transfer coefficient (*h*) of water is higher than that of air. Where the time of cooling of the temperature difference is inversely proportional to *h*. So, the percentage of *h*_water_ to *h*_air_ should be determined. By using Eq. (18) for the natural convection: *h*_water_ = 3.9 (≈ 4) *h*_air_.

Then, the times would be the time on air divided by 3.9 to get the time in the water:

- Initial temperature 120° to 25° : 40 /4 = 10 s.
- Initial temperature 55° to 25° : 12.63 / 4 = 3.16 ≈ 3 s.
- Initial temperature 40° to 25° : 6.32 / 4 = 1.58 ≈ 2 s.

For EV battery carrier in the air:

The time required for heat conduction through a plate is proportional to the square of the thickness, according to the transient heat conduction principle of the Fourier number, where it remains constant for similar temperature changes. The area does not affect the time for conduction through the thickness as long as the heat flow is one-dimensional, which is valid for plates with large surface areas compared to thickness.

26 $${\mathrm{Fourier number}} = {{\boldsymbol{\upalpha}}}t/T^{{2}}$$

where **α** is the conduction thermal diffusivity, ***t*** is the time, and ***T*** is the thickness.

- The ratio of thicknesses is ***T***_***EV***_ / ***T***_***Rov***_ = 2, So the time ratio is (***T***_***EV***_ / ***T***_***Rov***_)^2^ = 4.
- So, the required time for transferring heat from the upper to the lower plate is:
  $$t_{EV} = t_{Rov} \times { 4 } = { 4}0{\text{ s }} \times { 4 } = { 16}0{\text{ sec}}$$

Where the conduction is a linear equation and the time is proportional to temperature differences, the calculated times at 40° and 55° are:

- Initial temperature 120° to 25° : ΔT = 95 °C, time = 160 s.
- Initial temperature 55° to 25° : ΔT = 30 °C, time = (30 / 95) × 160 = 50.52 ≈ 51 s.
- Initial temperature 40° to 25° : ΔT = 15 °C, time = (15 / 95) × 160 = 25.26 ≈ 25 s.

##### Calculations of the ROV lower plate cooling time at different velocities

The common velocity of ROV is 2 m/s in subsea. The cooling time could be determined by Eqs. (24, 25) for both fluids, air and water, from velocity 0.5 m/s up to 2 m/s by step 0.5, as shown in Tables 3 and 4, where:

$${\mathrm{t}} = - \frac{2700 \times 0.125 \times 0.125 \times 0.001 \times 900}{{0.125 \times 0.125}}\mathop \smallint \limits_{{T_{i} }}^{{T_{f} }} \frac{dT}{{h { }\left( {{\text{T }} - 293} \right){ } + { }\left( {2.835 x 10^{ - 9} } \right) { }\left( {T_{s}^{4} { } - { }293^{4} } \right)}}$$

**Table 3:** Results of cooling time (t) in seconds of air fluid.

Air velocity	Parameters								
U_m_ (m/s)	T 40 °C			T 55 °C			T 120 °C		
	h_40_^o^ (W/m^2^ K)	Q˙_T_ (Watt)	t_T_ (sec)	h_55_^o^ (W/m^2 ^K)	Q˙_T_ (Watt)	t_T_ (sec)	h_120_^o^ (W/m^2^ K)	Q˙_T_ (Watt)	t_T_ (sec)
0.5	7.58	2.47	7:13	7.29	4.18	10:33	6.64	11.10	18:03
1	12.38	3.97	4:31	10.31	5.83	7:38	9.39	15.40	13:08
1.5	15.16	4.84	3:44	12.63	7.10	6:18	11.50	18.70	10:55
2	17.51	5.57	3:15	14.58	8.17	5:30	13.28	21.48	9:34
Film Temp. (T_film_)	30 °C			37.5 °C			70 °C

**Table 4:** Results of cooling time (t) in seconds of water fluid.

Air velocity	Parameters								
U_m_ (m/s)	T 40 °C			T 55 °C			T 120 °C		
	h_40_^o^ (W/m^2^ K)	Q˙_T_ (Watt)	t_T_ (sec)	h_55_^o^ (W/m^2 ^K)	Q˙_T_ (Watt)	t_T_ (sec)	h_120_^o^ (W/m^2^ K)	Q˙_T_ (Watt)	t_T_ (sec)
0.5	29.51	9.32	1:55	28.40	15.72	2:48	25.86	41.14	4:48
1	48.22	15.17	1:11	40.17	22.16	2:00	36.58	57.88	3:27
1.5	59.06	18.56	0:59	49.20	27.09	1:38	44.80	70.73	2:51
2	68.19	21.41	0:51	56.81	31.26	1:26	51.73	81.56	2:30
Film Temp. (T_film_)	30 °C			37.5 °C			70 °C		

**Fig. 10:**
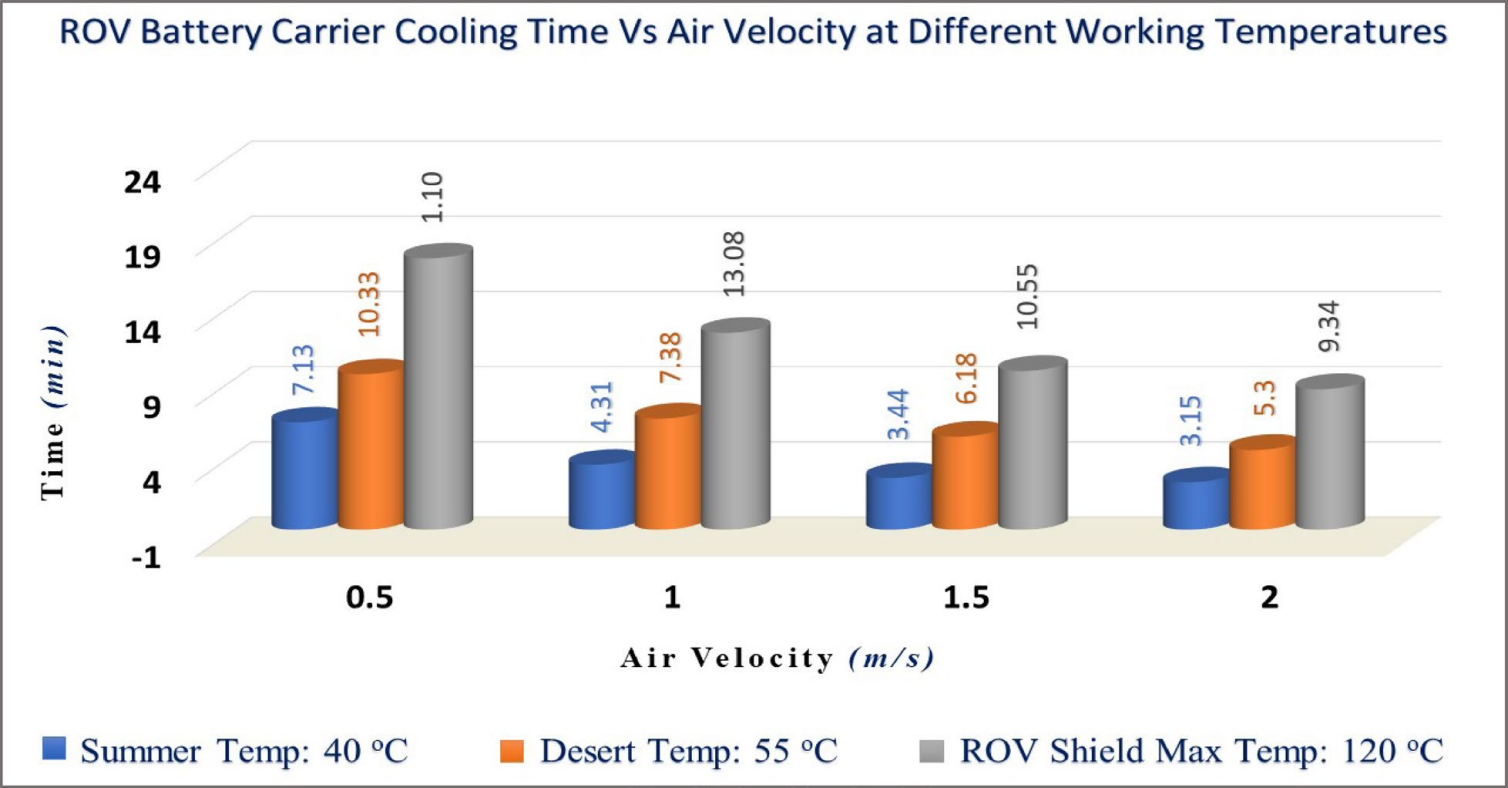
Cooling time of ROV battery carrier versus air fluid velocities (0.5:2 m/s).

**Fig. 11:**
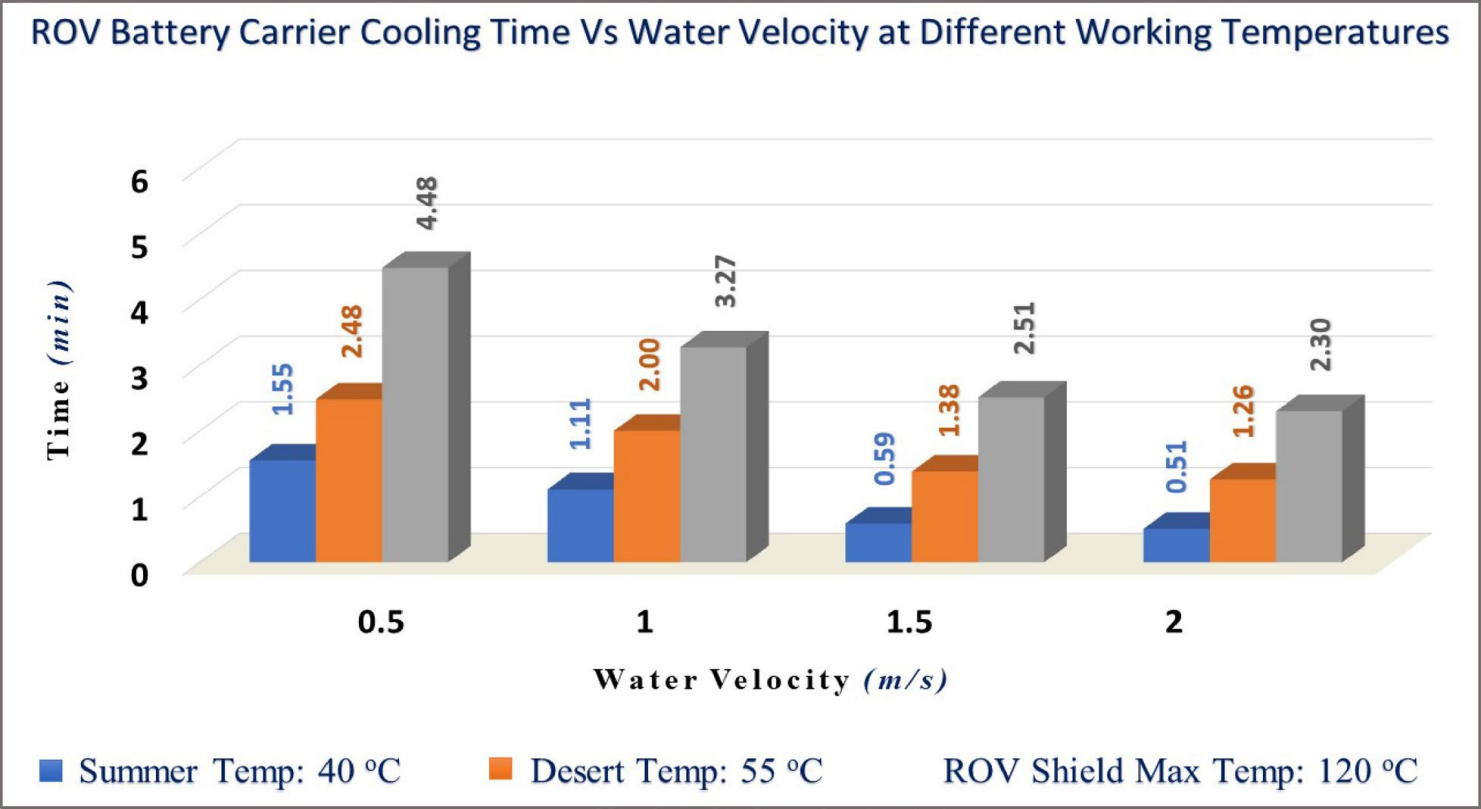
Cooling time of ROV battery carrier versus water fluid velocities (0.5:2 m/s).

The results indicate the required time for cooling of the ROV battery carrier’s lower plate, which faces the fluid. Figures 10 and 11 display a graph of cooling time versus different velocities from 0.5 to 2 m/s (1.8 to 7.2 km/hr) for both air and water fluids.

##### Calculations of the EV lower plate cooling time

**Fig. 12:**
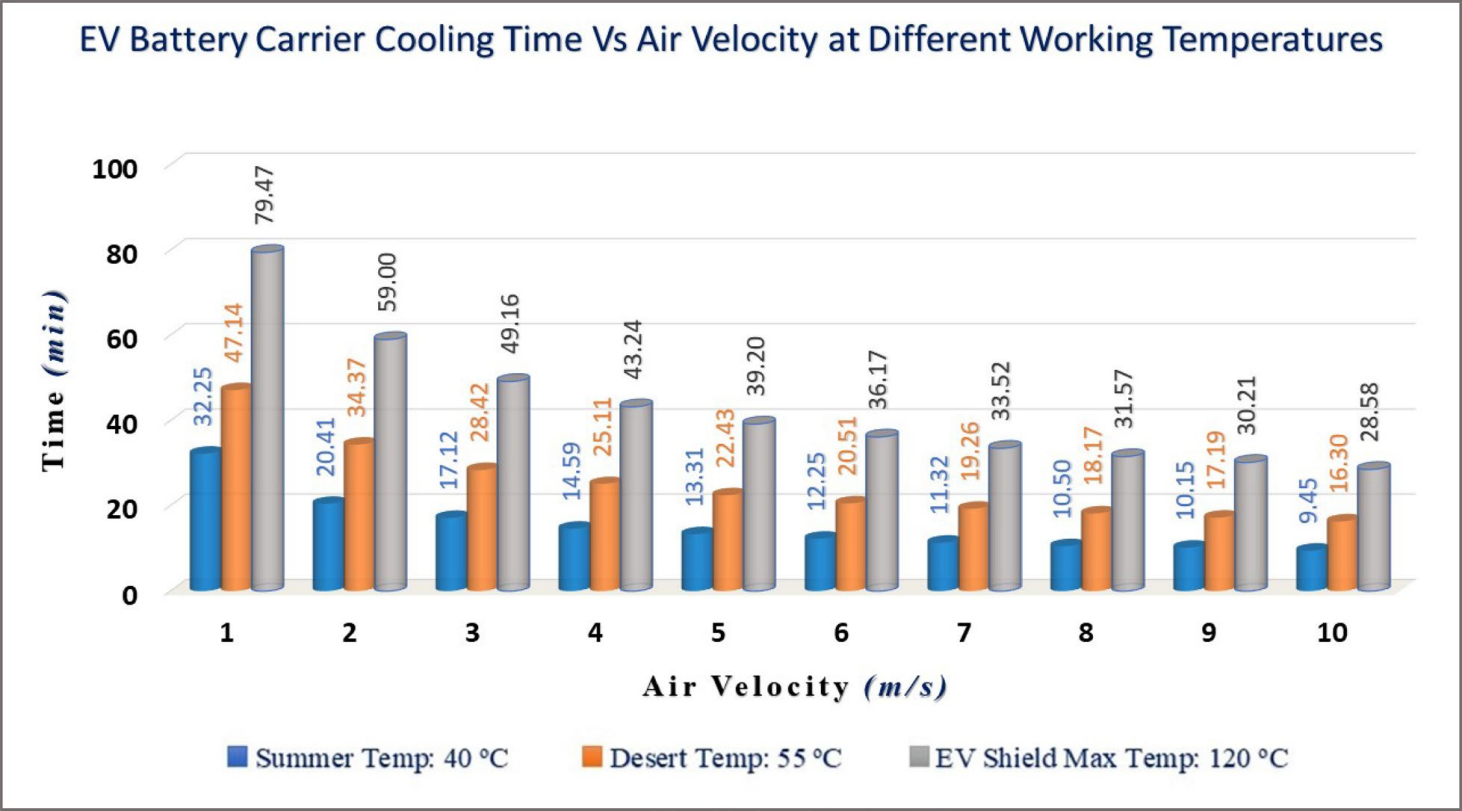
Cooling time of EV battery carrier versus air fluid velocities (1:10 m/s).

The results of the heat transfer coefficient, heat dissipation energy, and cooling time at air velocities from 1 to 10 m/s are displayed in Table 5. The results indicate the required time for cooling the EV carrier’s lower plate. Figure 12 displays the graph for the required time for cooling the shield versus different velocities from 1 to 10 m/s (3.6 to 36 km/hr). Note that the required time for transferring heat from the upper plate to the lower plate through blocks is estimated to be around 40 s at a maximum of 120 °C at the start of the moving car at an air velocity of 1 m/s and ambient temperature of 20 °C as the assumptions of the ROV battery carrier. The EV battery carrier is a large-scale pattern relative to the ROV battery carrier. The velocity of an EV can reach up to 60 m/s on the road. The cooling time could be determined by Eqs. (24, 25) for air fluid at low velocities from 1 m/s up to 10 m/s by step 1, as shown in Table 5. Where:${\mathrm{t}} = - \frac{2700 \times 1.5 \times 1 \times 0.002 \times 900}{{1.5 \times 1}}\mathop \smallint \limits_{{T_{i} }}^{{T_{f} }} \frac{dT}{{h { }\left( {{\text{T }} - 293} \right){ } + { }\left( {2.835 \times 10^{ - 9} } \right) { }\left( {T_{s}^{4} { } - { }293^{4} } \right)}}$

**Table 5:** Results cooling time (t) in seconds of air fluid for velocities from 1 to 10 m/s.

Air velocity	Parameters								
U_m_ (m/s)	T 40 °C			T 55 °C			T 120 °C		
	h_40_^o^ (W/m^2^ K)	Q˙_T_ (Watt)	t_T_ (sec)	h_55_^o^ (W/m^2 ^K)	Q˙_T_ (Watt)	t_T_ (sec)	h_120_^o^ (W/m^2^ K)	Q˙_T_ (Watt)	t_T_ (sec)
1	3.21	105	32:25	3.09	180	47:14	2.81	492	79:47
2	5.24	166	20:41	4.36	247	34:37	3.97	666	59:00
3	6.42	202	17:12	5.35	299	28:42	4.87	800	49:16
4	7.41	231	14:59	6.17	342	25:11	5.62	913	43:24
5	8.28	258	13:31	6.90	380	22:43	6.28	1013	39:20
6	9.08	281	12:25	7.56	415	20:51	6.88	1103	36:17
7	9.80	303	11:32	8.17	447	19:26	7.44	1185	33:52
8	10.48	323	10:50	8.73	476	18:17	7.95	1262	31:57
9	11.11	342	10:15	9.26	504	17:19	8.43	1335	30:21
10	11.72	360	9:45	9.76	530	16:30	8.89	1403	28:58
Film Temp. (T_film_)	30 °C			37.5 °C			70 °C		

## Evaluation of the design of EV and ROV battery carrier and challenges

**Fig. 13:**
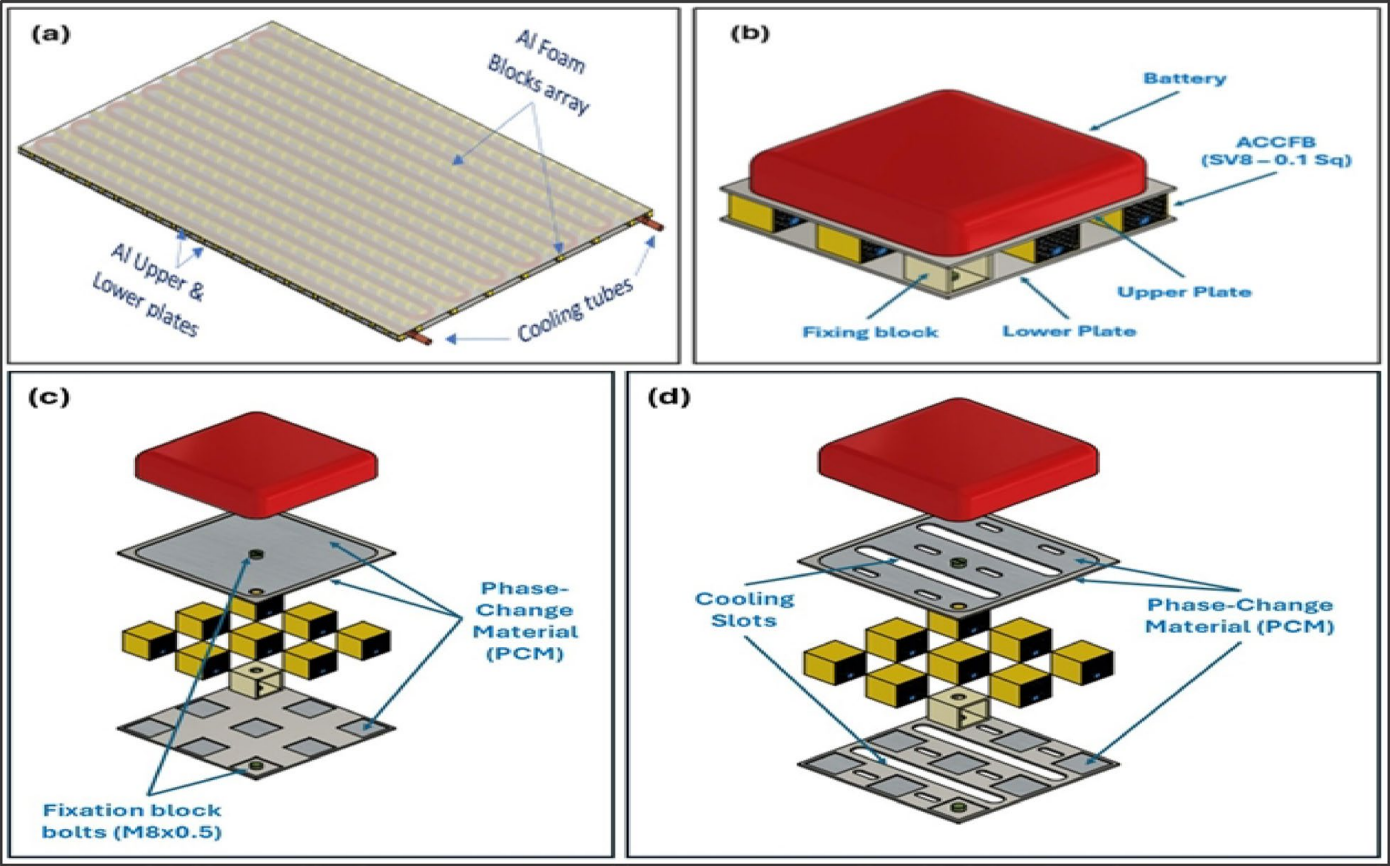
Schematics for the developed ideas of EV and ROV battery carriers: (**a**) carrier provided with a liquid cooling tube, (**b**) Battery carrier components, (**c**) Battery carrier supported by PCM, (**d**) Battery carrier supported by cooling slots.

The shapes of aluminum closed-cell foam blocks (ACCFBs) represent a broad concept, distinguished by their flexibility and ability to incorporate other design ideas. So, 1- For liquid cooling: the research team developed a simple idea for enhancing cooling (still under study), for providing the same EV carrier with a cooling tube system. Figure 13-a displays a preliminary schematic for this idea to suit hot environments. 2- Saving the cooling stability by using Phase change materials (PCMs), where it can be put between the battery’s lower outer surface and the outer surface of the upper plate of the carrier. Figure 13-b shows the Battery carrier components. Also, it can be put between the block’s upper and lower surfaces and the outer surfaces of the upper and lower plates, as appears in Fig. 13-c. 3- For the slotted AFS used in modern Al crush structure, a thick plate of foam is used (about 50–60 mm) to increase the energy absorption to compensate for the loss of energy absorption in the slotted areas. In EV and ROV carrier designs, you can arrange the position and shape of the distribution of blocks to open slots without needing to change the safety factor, as shown in Fig. 13-d. Increasing the thickness of the foam outside the design limits will reflect on reducing ground clearance or raising the car height; it will require modifying the design of the vehicle’s suspension system or aerodynamics.

### Achievements and challenges

ACCFBs were introduced into battery carrier design to solve the issue of wire harness intersections in remote operating vehicles. A primary challenge was that safety requirements for shock protection often conflicted with cooling system demands. The new design addresses this by incorporating specific, dedicated pathways for cables, allowing them to be routed without interference or path changes. Furthermore, the design provides effective heat dissipation for the energy generated by the wires while also protecting the shielded batteries from shocks by absorbing impact energy with equal or higher efficiency.

*The key design achievements were:* 1- Easy manufacturing and maintenance due to this design. 2- A static load capacity that exceeds the dynamic load, as per fundamental design principles. 3-Safety factors that satisfied all required load conditions. 4- The easy method for calculating the total impact force and impact stress. 5- Cooling time estimation achieved perfectly.

*The design challenges were:* 1-Applying a dynamic impact test to avoid the use of an excessively high safety factor, which would negatively impact the equipment’s weight, manufacturing cost, and maintenance. 2-Conducting a damping test to calculate the total damping of the carriers. 3- Performing a fatigue test to determine the endurance of the blocks and, consequently, the endurance limit of the carriers.

All these tests should be studied in future research to develop a comprehensive understanding of the mechanical properties of ACCFBs. This will lead to the design of carriers based on clear rules, eliminating the need for further assumptions.

## Conclusions

- Al closed-cell foam blocks (ACCFBs) are produced in limited volumes (in^3^) as an alternative to larger Al foam plates. This approach enhances energy absorption and addresses issues such as the manufacturing of nonuniform parts, high maintenance costs, and excessive thermal insulation.
- Working medium and the natural potential of selected material for designed carriers are important to avoid Galvanic corrosion especially with amphoteric material like Al.
- The ROV battery carrier is classified as low load, so the energy absorption is 243 J, **δ**_allowable_: 1.64 MPa, **δ**_working_: 1.093 MPa, and designed crushing load F_crush_: 13.9 kN at selected S.F by 1.5 due to light load. While the EV battery carrier is classified as medium load, so the energy absorption is 10.8 kJ, **δ**_allowable_: 2.47 MPa, **δ**_working_: 0.516 MPa, and designed crushing load F_crush_: 617 kN. at designed S.F: 4.8 due to battery load (S.F selected from 4 to 6 for crashworthiness).
- Cooling time of forced convection depends on material, shape, dimensions, and the type of fluid. Hence, the calculated total time of cooling for the conduction and forced convection for both the ROVs and EVs carriers at a cooling temperature of 25 ºC and at air velocity 1 m/s were 4:31 min and 32:25 min. At 2 m/s were 3:15 min and 20:41 min, respectively, at a summer working temperature of 40 ºC (Normal condition).
- Future research should study the dynamic impact, Damping, and Fatigue tests of ACCFBs to develop a comprehensive understanding of their mechanical properties. This will lead to the design of carriers based on clear rules, eliminating the need for further assumptions or excessive safety factors.

## Data Availability

All data generated or analyzed during this study are included in this published article.
